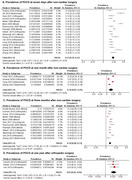# Prevalence of postoperative neurocognitive disorders in older non‐cardiac surgical patients: a systematic review and meta‐analysis

**DOI:** 10.1002/alz70860_097007

**Published:** 2025-12-23

**Authors:** Wendy WY Huang, Shirley Fan, Wei‐Ya Li, Vetri Thangavelu, Aparna Saripella, Marina Englesakis, Ellene Yan, Frances Chung

**Affiliations:** ^1^ School of Medicine, University of Limerick, Limerick, Munster, Ireland; ^2^ Schulich School of Medicine and Dentistry, Western University, London, ON, Canada; ^3^ Toronto Western Hospital, University Health Network, University of Toronto, Toronto, ON, Canada; ^4^ Temerty Faculty of Medicine, University of Toronto, Toronto, ON, Canada; ^5^ Toronto Western Hospital, University Health Network, Toronto, ON, Canada; ^6^ University Health Network, Toronto, ON, Canada

## Abstract

**Background:**

The growing number of older persons undergoing surgery are at a higher risk of neurocognitive disorder due to multimorbidity and age‐related changes. The prevalence of postoperative neurocognitive disorder in this population requires further investigation. This systematic review and meta‐analysis aims to estimate the pooled prevalence of perioperative neurocognitive disorder in older non‐cardiac surgical patients.

**Method:**

A comprehensive search of multiple databases was conducted from inception to January 24, 2024. This review included studies of non‐cardiac surgical inpatients aged ≥60 years old who underwent perioperative cognitive assessments. The primary outcome was the prevalence of postoperative neurocognitive disorder or cognitive dysfunction (POCD). Data were analyzed using a random‐effects model to calculate pooled prevalence rates. Quality assessment employed the Newcastle‐Ottawa Scale and MOOSE guidelines. Meta‐regression was performed with Open Meta Analyst and RStudio 4.3.3.

**Result:**

Thirty‐nine studies (*n* = 12,921) were included with mean age of 70.0 ± 8.9 years and 44.3% women. The overall prevalence of POCD was 23% (95% CI: 20%, 27%) at day 7, 16% (95% CI: 7%, 25%) at 1 month, 10% (95% CI: 8%, 13%) at 3 months and 3% (95% CI: 2%, 4%) at 1 year (Figure 1). Our meta‐regression showed a higher prevalence of POCD in abdominal surgery at day 7 (β = 0.13, 95% CI: 0.03–0.22, *p* = 0.01) and 3 months (β = 0.49, 95% CI: 0.40–0.58, *p* < 0.001), versus orthopedic surgeries.

**Conclusion:**

The overall prevalence of POCD in older non‐cardiac surgical populations was 23%, 16%, 10%, and 3% at day 7, 1 month, 3 months, and 1 year, respectively. Abdominal surgery had a higher prevalence of POCD than orthopedic surgery. The significant risk of POCD calls for cognitive screening, risk mitigation and intervention to provide better perioperative care and improve surgical outcomes.